# Interplay Between Brain Dominance, Reading, and Speaking Skills in English Classrooms

**DOI:** 10.3389/fpsyg.2022.798900

**Published:** 2022-03-16

**Authors:** Shanshan Li, Waode Hanafiah, Afsheen Rezai, Tribhuwan Kumar

**Affiliations:** ^1^Sanquan College of Xinxiang Medical University, Xinxiang, China; ^2^English Education Department, University of Dayanu Ikhsanuddin, Bau-Bau, Indonesia; ^3^Department of Teaching English and Linguistics, University of Ayatollah Borujerdi, Borujerd, Iran; ^4^Department of English Language and Literature, Prince Sattam Bin Abdulaziz University, Al-Kharj, Saudi Arabia

**Keywords:** brain dominance, left-right hemisphere, reading skill, speaking skills, speaking accuracy, fluency and comprehensibility

## Abstract

One of the popular theories in psychology that potentially contributes to the development of teaching and learning programs is brain dominance. According to this theory, the brain is categorized into two hemispheres based on personal traits and cognitive styles. It is interesting to investigate the correlation between brain dominance and second language learning. Therefore, this study set out to examine the correlation between brain dominance and the development of English reading, and speaking skills. For this purpose, the required data were randomly gathered from 230 sophomore students in four different universities and were analyzed through a Pearson Chi-Square test, a Kruskal–Wallis test, and a Mann–Whitney test. Findings evidenced a significant correlation between brain dominance and reading skills. Three categories of brain dominance groups differ in reading skills in which moderate right-brain shows the highest score. Concerning the speaking skills, however, the results documented no significant correlation between brain dominance and speaking skills. Three groups of brain dominance were not significantly different in three aspects of speaking skills, including accuracy, fluency, and comprehensibility. The study concludes by proposing a range of implications and some avenues for further research.

## Introduction

English has been an essential subject of educational tradition in Indonesia, taught as a foreign language. To maximize the transformation of English in the teaching and learning process, studies of classroom interaction, teaching methodologies, materials, learning approaches, learning styles, students’ behaviors, and other aspects are conducted to provide theoretical and practical contributions (Lightbown and Spada, 2021). As a part of psychology, brain and individual behaviors play an essential role in achieving educational objectives. One of the popular theories in neurology that is correlated with teaching and learning processes is brain dominance (Kök, 2014). This theory is concerned with the classification of the human brain into two hemispheres based on personal traits and cognitive style. It is deemed that each individual is different in terms of doing specific tasks (Niknam and Saberi, 2017; Seneviratne et al., 2019).

The fundamental question is why the study of the human brain is important in education. The reason for this as Hughes et al. (2017) note, is that the human brain is the center of processing information that works based on the specialization of structure and its function. The brain organ is the most complicated inter-connection cell that consists of 10 billion nerve cells (neurons) and billions of fibers to connect these cells (Steinberg, 2005; Namaziandost et al., 2018). In correlation between brain and education, Hart (1983) states that teaching without understanding how the brain works can be more or less similar to designing a glove with no sense of what hand looks like. In education, classrooms are places of learning and thinking; the brain is an organ of the mind.

It is rational to imagine that one of the critical factors in storing and recalling new information in memory is learning styles, in general, and brain dominance, in particular (Hughes et al., 2017). The reason for this is that “learners have clear preferences for how they go about learning new material” (Lightbown and Spada, 2021, p. 58). The previous studies have lent credence to the fact that L2 learners could benefit from the instructions which fitted into their cognitive preferences compared to the instructions which overlooked their hemispheric presences (Tendero, 2000; Saleh, 2001; Alibeigi, 2017; Vadivu and Chupradit, 2020). For example, Tendero (2000) reported that L2 learners’ brain dominance is a strong predictor of the development of L2 reading and writing skills. Additionally, Alibeigi (2017) reported that the brain dominance factor was significantly correlated with Iranian EFL learners’ vocabulary retention. Concerning the learning of the first language in the elementary cycle, teachers usually prioritize the analysis and synthesis of language components which is in favor of left-brained-oriented students. On the other hand, as Oflaz (2011) notes, when the educational programs put creativity first, the right-brained-oriented students take more advantage of the instructions.

Though in the literature, a range of studies (Oflaz, 2011; Kök, 2014; Alibeigi, 2017; Niknam and Saberi, 2017; Wei et al., 2017) has addressed the correlation between brain dominance and L2 learning in other countries, this domain has remained unexplored in the Malaysian context. It is essential to disclose the correlation of brain dominance with the learning of L2 skills is. Therefore, the present study is an attempt to bridge the gap in the literature and further our understanding of the correlation between brain dominance and the learning of L2 reading skills and speaking skills. The findings of the present study may be helpful for L2 teachers to make them aware of the role of brain dominance in L2 learning, and, accordingly, help them adapt their ways of teaching L2 learners’ cognitive styles to improve substantially their reading skills and speaking skills. Further, materials developers may benefit from the results of this study to take into account the role of the learners’ cognitive styles prior to starting the development of materials.

## Review of Literature

Calculating the brain dominance concept in English teaching could clarify that the personality type of brain dominance may affect learning outcomes of learners’ receptive and productive skills. Tendero (2000) studied the correlation between learners’ hemispheric dominance and their language proficiency levels of four macro language skills at Western Mindanao State University in two groups of learners; age classification and gender. The study revealed that left-brain students scored the right-brain and the whole brain in speaking tests for a group of 16 years old and below. For the 17 and 18 years old, the right and entire brain students got higher scores than the left-brain ones in the reading test. For groups 19 and 20 years old, left-brain students got higher scores than the right and whole brains in listening and speaking tests and in the global English proficiency test. Based on gender, brain dominance did not significantly influence both male and female students’ listening, speaking, reading, and writing. Khabiri and Heidari (2011) studied the relationship among EFL learners’ left-right-brain dominance, autonomy, and reading comprehension of the academic and general reading modules of the International English Language Testing System (IELTS). They found that there was a significant correlation between the two studied variables. Oflaz (2011), in the Turkish context, found that right-brain students were better at responding, demonstrating instruction, and visual performance in the part of the vocabulary. In the writing part, they were also better at the open-ended question. The left-brain students who were good at problem-solving by logic were better at speaking English and reading piece. The previous study of brain dominance and English skills shows that brain dominance influences the output of students’ competence in reading and speaking. In the Indonesian context, where English is taught as a foreign language, this current study of brain dominance on speaking skills shows no significant correlation between the two variables.

Association of the brain and individual preference has been observed for many years. In 1946, Sperry found that the brain function mechanism of the corpus colosseum, the concept of split-brain, represented the significant set connection between two cerebral hemispheres in which speech interpretation and production center were located in the left side (Voneida, 1997). Concerning brain and language, Chomsky (2006) states that three components of the biological system contribute to individual language development. They are “genetic factors, experience, and principle not specific to the faculty of language.” Human brain activation is potentially influenced by genetic factors, from which biologically neural circuitry of brain cells determines individuals’ language development and performance. Brain mechanism results in language instinct in the human mind to perform language competence from which an innate linguistic knowledge produces “universal grammar.” Chomsky, however, does not specify that parts of the human brain function to process language mechanisms. He focuses on “language competence or knowledge of language” rather than how the language is processed in the human brain. His claim differs from what Saussure (1959) states, that the real purpose of research in the linguistic field is to discern phenomena related to language knowledge (“la langue”) and “extraneous events (parole).” Understanding grammatical rules or language construction correlated to human experience influences language production and perception. The fact, however, is ignored that a complex neurological system contributes to how language is performed. Lieberman (2000) states that “language is a learned skill” controlled by “a functional language system (FLS)” through the distribution of physical activity in many parts of the complex human brain. FLS functions to regulate the circulation of language production and perception, which exists in the only human brain. It connects to “other aspects of cognition, motor control, and emotion.”

## Theoretical Framework

Brain functioning and hemisphericity are two crucial characteristics of learner differentiation in language learning. The hemisphericity theory, which has gained popularity in recent years, proposes that individuals choose a preferred mode of cognitive processing linked to action on behalf of the left or right hemisphere (Bavand Savadkouhi et al., 2013). Further, Alptekin and Atakan (1990) interpret hemisphericity as a person’s inclination to believe one cerebral hemisphere more than the other, regardless of the attitude and mindset of task requirements. Hemisphericity was described by Leng et al. (1998) as “the propensity for using one side of the brain more than the other.” (pp. 115–116).

The study of Hudson (2000) also mentioned brain hemisphericity as right, left, or complete brain dominance. He discovered that the left hemisphere is stronger at analytical and temporal tasks like mathematics, riddles, music, and alphabetic reading. Likewise, Stevick [as quoted in Brown (2000)] concluded that left-brain-dominant second language learners appeared to be competent at producing independent words, gathering linguistic characteristics, performing function cycles, and using conceptualization, categorization, naming, and restructuring. On the other hand, right-brain dominant individuals appear to function effectively with full imagery and generalization, metaphors, emotive input, and artistic and spectacular claims (Brown, 2000). The right hemisphere analyzes non-verbal, tangible, and spatial information, pays attention to connections, and views everything holistically. As a result, the right brain is acknowledged to have a worldwide tendency. Other right-brain activities include creative abilities, such as music and graphics (Dulger, 2012; Gunasinghe et al., 2020; Hamid et al., 2020).

Although some people prefer to process information with their left- or right-brain, others may use both hemispheres simultaneously, benefiting the student in instructional procedures (Dulger, 2012). Entire-brained learners employ all of the tactics that right and left-brained learners employ (Morris, 2006; Namaziandost et al., 2020). It is important to note that the left and right hemispheres are not fully autonomous and function as a team, and it is the corpus callosum that connects both hemispheres (Saleh, 2001) to make the vital neurological connection of the brain for the optimal dilemma.

Several studies have been conducted to investigate the association between hemisphericity and language abilities, such as speaking (Mireskandari and Alavi, 2015; Namaziandost et al., 2019), listening (Kök, 2014), writing (Weisi and Khaksar, 2015), and reading (Weisi and Khaksar, 2015). (Kök, 2010; Khabiri and Heidari, 2011). Mireskandari and Alavi (2015) discovered a statistical difference in the use of compensating methods among whole-brain dominant respondents and both right and left-brain students. According to the research done by Weisi and Khaksar (2015), right-brain dominant students were more innovative in a writing exam. Kök (2014) discovered no significant difference in listening comprehension between control and experimental group participants in terms of hemisphere dominance.

Some researchers considered the possible relationship between hemisphericity and other characteristics and its influence on those parameters (e.g., Saleh, 2001; Dulger, 2012). Dulger (2012) used the Oxford (1990) strategy survey scale for language learning and the Davis et al. (1994) Brain Dominance Inventory to determine whether there is a correlation between brain dominance and language learning strategy utilization among university learners. Although metacognitive techniques were shown to be the most commonly employed technique (*M* = 3.77), the findings did not indicate that brain dominance was associated with metacognitive strategies.

The study conducted by Kök (2010) offers an in-depth examination of the relationship between learners’ use of reading comprehension strategies, their reading comprehension successes, and their perceptions of learning English concerning hemispheric dominance by drawing attention to the notion of hemisphericity and reading comprehension and other factors. He conducted a study of 40 respondents from a preparatory university program. According to the data analysis, there was no statistical difference in reading comprehension performance. Nevertheless, the findings revealed a statistically significant difference in perceptions between the experimental and control groups, favoring the experimental group in both left and right-brain dominant participants.

In a similar vein, Khabiri and Heidari (2011) investigated the association between left- and right-brain dominance, autonomy, and Efl Students’ reading comprehension of the Academic and General Reading Modules of IELTS. They led the research on 100 randomly chosen EFL students enrolled in IELTS preparation classes at a language institute in Tehran. As per the research, all participants were asked to complete the brain dominance questionnaire and learners’ autonomy survey, but 50 of them took the IELTS General, and the remaining 50 took the Academic Reading Module. The findings revealed that learner autonomy had no apparent link with attendant performance, while brain dominance had a relationship.

The theory of brain dominance classifies individual preference into two contrastive characteristics. Dubin (2001) states that brain cells are classified into two leading hemispheres with different and specific functions to respond to visual signals transmitted to the brain processor from the eyes. In particular brain function hemisphere, Steinberg (1993) states that the brain consists of specific structures and functions. The left hemisphere controls language, logical and analytical operation, and higher mathematics. In contrast, the right hemisphere is superior at recognizing emotions, recognizing faces, and taking in the structures without deep analysis. Contrastive brain works are also claimed by Schwartz (2010) that classify the different functions of the left- and right brain. The left-brain tends to be verbal, rational, quantitative, analytic, deductive, simplified, specialized, separated, critical, goal-oriented, sequential, systematic, objective, literal, rule-bound, and outcome-driven, while the right-brain tends to be visual, intuitive, qualitative, synthetic, inductive, enriched, integrated, connected, non-judgmental, big picture-oriented, simultaneous, emphatic, subjective, symbolic, unbounded, and process-driven.

Individuals’ cognitive styles associated with the brain hemisphere possibly derive from two main factors, heredity, and environmental condition. Some scientists of the genetics field assume that there is a possible correlation between the specific structure of two halves of the brain and enzymes production due to heredity factors (Gardner, 1993). Environmental differences contribute to the functions performed by the particular brain hemisphere area (Hall, 2005). Brain dominance to each individual can be changed depending on the routines that predominantly activate the left- and right brain. Goswami (2004) states that the brain, which is functioned to auditory analysis or hearing sounds, can serve as a visual or spatial analysis by deaf people who exercise sign language. Despite blind people who possess the dominant ability to hear due to heredity factors can develop performance to read and recognize objects forced by extreme environmental conditions. The purpose of this study is to find out the significant correlation between brain dominance and two English language skills, reading and speaking. Different categories of brain dominance are also analyzed and compared to find out specific differences and contributions of students’ preferences based on brain personality traits to reading as a receptive skill and speaking as a productive skill. The present study aims to answer the following research question:

Q: Is there any significant relationship between brain dominance and the development of reading and writing skills among Malaysian university students?

## Methodology

### Research Design

This research used a quantitative method, correlational study, to determine the degree of relationship between two variables, brain dominance, and two English skills reading and speaking. The first variable, brain dominance, was classified into five categories; left solid brain, moderate left-brain, middle brain, moderate right-brain, and strong right-brain. For the second variable, reading skill was determined by the students’ individual gained scores in the reading part of IELTS, and speaking skill was obtained from individual performance to present the ideas discussed. Data obtained from two variables were analyzed inappropriate test of International Business Machines Corporation (IBM) Statistical Package for the Social Sciences (SPSS, Version 20) to find out the significance in the correlation between two variables and the difference among categories of brain dominance. This research was conducted in the English department, from four different universities, the Alauddin Islamic State University of Gowa (hereafter is called AISUG), a state university and three private universities, Indonesian Muslim University of Makassar (IMUM), Muhammadiyah University of Makassar (MUM), and Cokroaminoto Unversity of Palopo (CUP), South Sulawesi, Indonesia.

### Participants

The population of this study consisted of the sophomore students of the English department in four different universities, specifically, 75 students of AISUG from two classes, 325 students of MUM from ten classes, 70 students of CUP, and 148 students of IMUM from five categories. The total population was 618 students. Using a random sampling method, 62 students from AISUG, 58 students from MUM, 70 students from CUP, and 40 students from IMUM were selected as the study sample. According to Riazi (2016), the random sampling method is used to give equal opportunity to the individuals in a population to be selected in a survey. As such, the study involved 230 students (37.2% of the total population). The primary reason for selecting the participants was their easy accessibility to the researchers.

### Techniques of Data Collection

Data were collected twice in different quantities. One hundred fourteen samples from three universities to obtain reading scores and 190 samples from three universities to obtain speaking scores. In the process of data collection, a brain dominance test was randomly distributed to the samples. The test was from the alert scale of cognitive style, designed by Crane (1989), from which he set the test that consisted of 21 questions. Each student was asked to choose one option of two options in each question. To avoid students’ misunderstanding of the meaning of the words on the test, the original test in the English version was translated into Indonesian. In doing the brain dominance test, the researcher clearly explained the meaning of items on the test to obtain accurate students’ brain dominance preferences.

The reading test (IELTS) was distributed to 114 selected students who had completed the brain dominance test to obtain students’ reading scores. In speaking, the selected samples, 190 were asked to perform speaking competence based on the discussed title. To find out students’ skills in speaking, they were asked to discuss in pairs related to their favorite country. In this session, the researcher distributed a small paper as a guide for students to speak. In the article, the researcher wrote four questions related to the discussed topic (Favorite country) those are (1) What is your favorite country? (2) To what aspects do you like in it (economy, people, politics, law, landscape, business, tourism, military, technology, science, education, entertainment, etc.)? (3) Why do you like those aspects? (4) If you have an opportunity to visit it, what will you do? Students were asked to present what they had discussed in pairs without reading individually in the last session. They were allowed to improvise items on the papers based on students’ prior knowledge. Their voice in speaking was recorded using an easy voice recorder, android program.

### Techniques of Data Analysis

Classification of students’ brain dominance adopted specific instruction on the source of the test from the alert scale of cognitive style, Western Michigan University, designed by Dr. Loren D. Crane in 1989. It consisted of 21 questions. One point was given to the respondents who answer “A” for number “1, 2, 3, 7, 8, 9, 13, 14, 15, 19, 20, 21” and answer “B” for number “4, 5, 6, 10, 11, 12, 16, 17, 18.” Then, the scores were computed to categorize hemispheric brain dominance based on the following classification:

0–4: Strong Left-Brain
5–8: Moderate Left-Brain
9–13: Middle-Brain
14–16: Moderate Right-Brain
17–21: Strong Right-Brain
[The Alert Scale of Cognitive Style by Crane (1989)]

The scoring system was adopted from the reading performance band score criteria from IELTS Essential (2015) and IDP education. The Scoring system of the speaking test was adopted measures of speaking standard introduced by Heaton (1988:100) that divided criteria into three aspects: accuracy, fluency, and comprehensibility. A student’s score of each item (accuracy, fluency, and comprehensibility) from three raters was converted into the following formula based on score classification:


$$\mathrm{Speaking}\,\mathrm{Score}=\frac{T\,h\,e\,G\,a\,i\,n\,S\,c\,o\,r\,e\,o\,f\,E\,a\,c\,h\,C\,r\,i\,t\,e\,r\,i\,a}{3}$$

Data obtained from the test were analyzed in IBM SPSS 20. To find out the Normality of the data, the One-Sample Kolmogorov–Smirnov Test, and the Shapiro–Wilk test were used. Since the output of the normality test showed that the data were not normally distributed, the Homogeneity test, Levene Test, and ANOVA were not used. To analyze the correlation between brain dominance and two skills of English, reading, and speaking, Pearson Chi-Square was used to find out the significant difference among different categories of brain dominance to reading skill, the Kruskal–Wallis and Mann–Whitney tests were used.

## Results

### Brain Dominance

**FIGURE 1:**
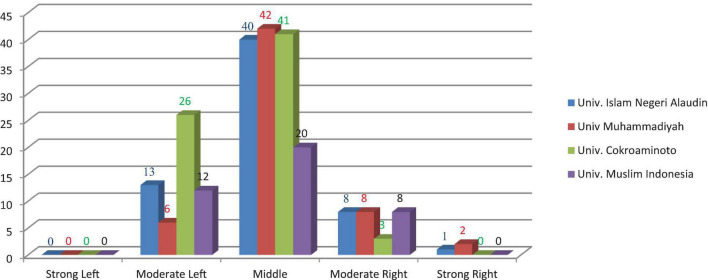
Histogram of brain dominance distribution.

The results of the brain dominance questionnaires reported that 230 samples, in AISUG, there were 13 students of the moderate left-brain (their scores ranged from 7 to 8), 40 students of the middle-brain (their scores ranged from 9 to 13), 8 students of moderate right-brain (their scores ranged from 14 to 15), and 1 student of strong right-brain (their scores ranged from 17 to 18). In MUM, there were 6 students of the moderate left-brain (their scores ranged from 6 to 8), 42 students of the middle brain (their scores ranged from 9 to 13), 8 students of moderate right-brain (their scores ranged from 14 to 16), and 2 students of strong right-brain (their scores ranged from 17 to 18). In CUP, there were 26 students of the moderate left-brain (their scores ranged from 5 to 8), 41 students of the middle-brain (their scores ranged from 9 to 13), and 3 students of moderate right-brain (their score was 14). In IMUM, 12 students of the rational left-brain (their scores ranged from 5 to 8), 20 students of the middle brain (their scores ranged 9 to 12), and 8 students of moderate right-brain (their scores ranged from 14 to 15). The total brain dominance score consisted of 57 students of the moderate left-brain, 143 students of the middle-brain, 27 students of the moderate right-brain, and only three students of the strong right-brain. From 230 samples, no one tended to the solid left-brain. The middle-brain dominated the distribution of brain dominance (62.2%), followed by moderate left-brain (24.8%), moderate right-brain (11.7%), strong right-brain (1.3 %), and strong left-brain (0%). The brain dominance distribution is presented in the following chart (Figure 1).

### The Correlation Between Brain Dominance and Reading Skill

Analysis of the Pearson Chi-Square test showed that the Chi-Square value was 15.239 at the degree of freedom 6, in which *P*-value (0.018) < 0.05. Therefore, it was turned out that there was a significant correlation between brain dominance and the IELTS reading skill. The output of the Pearson Chi-Square analysis is presented in Table 1.

**TABLE 1:** Results of Pearson Chi-square test.

	Value	Df	Asymp. Sig. (2-sided)
Pearson Chi-square	15.239	6	0.018
Likelihood ratio	16.782	6	0.010
Linear-by-linear association	5.720	1	0.017
N of valid cases	111		

### The Difference Among Categories of Brain Dominance to Reading Skill

Using statistical analysis to find significant differences among three groups of brain dominance to the reading skill of IELTS, the data were computed into normality test to decide appropriate SPSS test analysis. In this study, Kolmogorov–Smirnov and Shapiro–Wilk were used. The output of the normality test showed that in Kolmogorov–Smirnov, Moderate left-brain showed statistic value 0.406, *P*-value (0) < 0.05. It means that the moderate left-brain score of IELTS reading was not normally distributed. Middle brain showed that statistic value 0.251, *P*-value (0) < 0.05. It could be interpreted that the middle brain score of IELTS reading was not normally distributed. Moderate right-brain showed that statistic value 0.333, *P*-value (0) < 0.05. That is, the moderate right-brain score of IELTS reading was not normally distributed. In Shapiro–Wilk, Moderate left-brain showed statistic value 0.656, *P*-value (0) < 0.05. To put it in other words, the moderate left-brain score of IELTS reading was not normally distributed. Middle brain showed that statistic value 0.776, *P*-value (0) < 0.05. It means that the middle brain score of IELTS reading was not normally distributed. The moderate right-brain showed that statistic value 0.762, *P*-value (0) < 0.05. It showed that the moderate right-brain score of IELTS reading was not normally distributed. Since the Normality analysis indicated that the data of three brain dominance categories were not normally distributed, a non-parametric test was used. In this study, Kruskal–Wallis and Mann–Whitney were used. The output of the Kruskal–Wallis test (Table 2) showed that the Chi-Square value was 10.695, *P*-value (0.005) < 0.05. It can be said that there was a significant difference among the three categories of brain dominance to reading skills of IELTS.

**TABLE 2:** Results of the Kruskal–Wallis test.

	Reading score
Chi-square	10.695
Df	2
Asymp. Sig.	0.005

Mann–Whitney test was used as an alternative to *post hoc* analysis to analyze the significant difference among three groups of brain dominance. In comparison between moderate left-brain and middle brain, Mann–Whitney output showed that *Z*-value was −0.901, *P*-value (0.368) > 0.05. It means that there was no significant difference between moderate left-brain and middle brain concerning the reading part of IELTS. The difference between the moderate left-brain and moderate right-brain showed that *Z*-value was −3.101, *P*-value (0.002) < 0.05. It indicated that there was a significant difference between two categories of brain dominance, moderate left-brain and moderate right-brain regarding the reading part of IELTS. In the output of the difference between middle- and moderate right-brain, Mann–Whitney showed that *Z*-value was −2.798, *P*-value (0.005) < 0.05. It disclosed that there was a significant difference between the two categories of brain dominance, middle brain, and moderate right-brain with respect to the reading part of IELTS.

### The Correlation Between Brain Dominance and Speaking Skill

The analysis of Chi-Square showed that Pearson Chi-Square Value was 158,897 to the degree of freedom 165 at the level of significant 0.05. *P-*value was 0.681 > 0.05. It means that there was no significant correlation between hemispheric brain dominance and speaking skills. The results of the Chi-Square test are reported in Table 3.

**TABLE 3:** Results of Chi-Square test.

	Value	Df	Asymp. Sig. (2-sided)
Pearson Chi-square	158.897	168	0.681
Likelihood ratio	149.455	168	0.845
Linear-by-linear association	0.127	1	0.721
N of valid cases	182		

### The Difference Among Different Categories of Hemispheric Brain Dominance

Using SPSS version 20, the collected data were analyzed to reveal if there was a significant difference among three different categories of brain dominance. Prior to proceeding with this, the researchers examined if the collected data were appropriate for parametric or non-parametric. For this purpose, they examined the normality and homogeneity assumptions. Concerning the normality assumption, the Kolmogorov–Smirnov test and Shapiro–Wilk test were used.

The results of the Kolmogorov–Smirnov test revealed that moderate left-brain statistic was 0.069, degree of freedom was 45, and *P-*value was 0.2 > 0.05. It means that the data collected for the speaking skills for the moderate left-brain were normally distributed. To the middle brain, the statistic value was 0.163, degree of freedom was 124, and *P-*value was 0 < 0.05. It indicated that the data collected for the speaking skills for the middle brain was not normally distributed. To the moderate right-brain, the statistic value was 0.219, degree of freedom was 19, and *P-*value of 0.017 < 0.05, which means that the data collected for the speaking skills for the moderate right-brain was not normally distributed.

The results of the Shapiro–Wilk test displayed that the moderate left-brain statistic was 0.977, degree of freedom was 45, and *P-*value was 0.503 > 0.05. In other words, the data collected for the speaking skills of the moderate left-brain was normally distributed. To the middle brain, the statistic value was 0.949, degree of freedom was 124, and *P-*value was 0 < 0.05. That is, the data collected for the speaking skills of the middle brain was not normally distributed. Finally, to the moderate right-brain, the statistic value was 0.883, degree of freedom was 19, and *P-*value was 0.024 < 0.05. It demonstrated that the data collected for the speaking skills of the moderate right-brain was not normally distributed.

Having assured that the collected data were not normally distributed, the researcher did not check out the homogeneity assumption. Therefore, they used the Kruskal–Wallis test, as a non-parametric test. In the output of the Kruskal–Wallis test, the Chi-Square value was 0.487, the degree of freedom was 2, and *P-*value was 0.784 > 0.05. It means that there was no significant difference among different categories of brain dominance. The results of the Kruskal–Wallis test is presented in Table 4:

**TABLE 4:** Results of Kruskal–Wallis test analysis.

	Speaking competence
Chi-Square	0.487
Df	2
Asymp. Sig.	0.784

### The Difference Among Categories of Brain Dominance to Speaking Skill

To specifically analyze the level of significant difference between the moderate left-brain and middle-brain, the moderate left-brain and moderate right-brain, and the middle-brain and moderate right-brain, two independent samples *t*-tests were run used. The output of the Mann–Whitney test revealed that the speaking skills’ value was 2,638, *P-*value was 0.587 > 0.05. This means that there was no significant difference between the moderate left-brain and middle-brain. The difference between the moderate brain and left-brain output of the Mann–Whitney test displayed that the speaking skills value was 382, *P-*value was 0.502 > 0.05. That is, there was no significant difference between the moderate left-brain and moderate right-brain. The middle brain and moderate brain indicated the same findings in which the speaking skills value was 1,064.5, *P-*value was 0.858 > 0.05. To put in other words, there was no significant difference between the middle brain and the moderate right brain.

## Discussion

The research question investigated if there was any significant correlation between brain dominance and the development of English reading, and speaking skills. The findings evidenced that brain dominance was significantly correlated with their reading skills. The study’s findings are in line with those of Alibeigi (2017), reporting that brain dominance was a determining factor in Iranian EFL learners’ vocabulary learning and retention. However, the results of the study are partially in contrast with those of Kök (2014), indicating that there was not any meaningful correlation between EFL learners’ listening comprehension and their hemispheric dominance.

The findings indicated that the different categories of the students’ brain dominance preferences significantly contributed to their reading skills where the right-brain-oriented students outperformed the other students. To recap the discussion, the findings may be explained from the brain dominance theory. In line with the study’s findings, it may be argued that left-brain, and right-brain-oriented students may be good at handling different tasks. Along with Brown (2000), it may be argued that the left-brain-oriented students might have been more analytical, planned, and structured in the reading tasks, while the right-brain-oriented students might have tended to be synthesized, fluid, and spontaneous.

Regarding speaking skills, the results documented that there was no significant correlation between brain dominance and the participants’ speaking skills. According to the findings it may be discussed that the different cognitive styles and personality traits did not significantly influence the students’ performance on speaking tasks, measuring three criteria: fluency, accuracy, and comprehensibility. To discuss the findings, along with McGilchrist (2009), it may be argued that the specific classification between two hemispheres was overgeneralized since the brain function might have involved a complex interaction among distinct sides of the brain. The findings are also argued from this perspective that the activities in the left and right brain might have been the specific mental process that connected each other in which both hemispheres might have inter-connectedly conveyed and transmitted the information.

Concerning speaking skills, along with the brain dominance theory, it may be argued that the left hemisphere dominated the production of language. As Weaver (2013) stated, the speech patterns might have involved complex hierarchical components that might have occurred at different times in oral communication production. Both sides of the brain might have been active in producing and delivering the required information. Therefore, it might potentially result in the same contribution to the left and right hemispheres in the speaking activities. Thornbury (2005) states that L1 and L2 speakers have almost the same way to speak in terms of mental processing, starting from conceptualizing, then formulating, and then articulating. All stages involve self-monitoring. L1 and L2, however, differ in words of language and knowledge. Mother tongue vocabulary, grammatical rules, and understanding of the issue elaborated by the speaker can influence speaking ability.

These disparities in this study can be attributed to the fact that speaking and reading techniques provide students with the tools they need to perceive and generate language despite their low language expertise. This means that students may make informed guesses using both verbal and non-linguistic cues. Brown (2007) also said that, while language processes appear to be controlled by the left hemisphere, there is considerable right hemisphere involvement, which comprises acquisition techniques. Right hemisphere activity is characterized by techniques, such as guessing at meanings and employing formulaic speech (Obler, 1981). Because speaking is both verbal and non-verbal, and speaking techniques are a mixture of both verbal and non-verbal techniques, whole-brain dominant participants may have used these techniques more than the others because they were capable of utilizing both verbal and non-verbal behaviors simultaneously.

The findings support Yeap’s (1989) contention that neither left nor right hemisphere users are better or worse than the other and that their variances are simply due to the sorts of new data. In other words, the utilization of both the left and right hemispheres are equally viable ways of responding, interpreting, perceiving, and storing information, and they complement each other. According to Brown (2002), we can argue that the students’ dominance of the left or right hemisphere of the brain might have caused specific changes in their learning outcomes. According to the findings, it may be discussed that the left-brain-oriented students might have been more intellectual, remembered the names, reacted appropriately to verbal commands and interpretations, and therefore, performed better on the reading comprehension tests. To discuss the findings more, according to the findings it may be argued that the right-brain-oriented students might have used intuition and worked with hunches. They might have used their imagination and were strong with time conception. They might have had a tendency to see more of the whole. All this might have helped the student perform better on the reading comprehension tests.

## Conclusion

This study purported to investigate the correlation between brain dominance and the learning of English reading skills and speaking skills. The findings indicated that there was a significant correlation between the participants’ brain dominance and their reading skills. However, the results evidenced that brain dominance is not significantly correlated with the participants’ speaking skills. In light of the findings, it can be concluded that brain dominance is a crucial factor affecting L2 learners’ storing and recalling information. This effect is more noticeable in reading comprehension where L2 needs to receive input and accommodate it with the previous information to reach a correct understanding of the passage. In other words, the study’s findings lend credence to the words of Lightbown and Spada (2021) who succinctly put it: “learners have clear preferences for how they go about learning new material” (p. 58).

The findings of the present study offer some notable contributions. They shed light on the significance of brain dominance as a crucial cognitive factor that has a direct impact on L2 learners’ achievement. Furthermore, they could further the understanding of L2 practitioners concerning the importance of brain dominance in the development of L2 learning in the EFL contexts. As Wong and Nunan (2011) note, this understanding encourages L2 teachers to use learning approaches and procedures that are adopted to the cognitive learning preferences of their students. Additionally, L2 teachers could devise exercises that cater to both left- and right-brain students, utilizing the traditional loops, verbal model, and engaged, image-rich, visuospatial models, allowing students to use both hemispheres. Implementing teaching strategies that meet the requirements of all learners may be challenging, but if teachers evaluate their learners’ learning styles and balance their teaching by using a range of assignments in the classroom, they can reach success in this respect (Jhaish, 2010). Moreover, the study’s findings can be of great help to teacher educators. They can accommodate the information about brain dominance in their courses such that teacher students can gain clear insights into the issue. Likewise, the research findings can be helpful for materials developers. They should consider the fact that learners have different cognitive styles. Some learners are left-brain dominated and others are right-brain dominated. So, the materials should be designed in such a way that the learning needs of all learners can be met well.

Given the limitations imposed on this study, some avenues for further research are presented. First, the interested researchers can conduct similar studies utilizing a larger sample of sophomore students in other parts of the country. They can increase the generalizability of the present study’s findings. Second, as the present study was quantitative, future research can use qualitative design to disclose EFL learners’ perceptions of the role of brain dominance in their learning. Third, more studies are required to examine the effects of different teaching approaches and strategies on EFL learners’ brain dominance. Last but not least, future studies need to explore the correlation of brain dominance with the components of communicative competence, such as inter-language pragmatic competence.

## Data Availability Statement

The raw data supporting the conclusions of this article will be made available by the authors, without undue reservation.

## Author Contributions

All authors listed have made a substantial, direct, and intellectual contribution to the work, and approved it for publication.

## Conflict of Interest

The authors declare that the research was conducted in the absence of any commercial or financial relationships that could be construed as a potential conflict of interest.

## Publisher’s Note

All claims expressed in this article are solely those of the authors and do not necessarily represent those of their affiliated organizations, or those of the publisher, the editors and the reviewers. Any product that may be evaluated in this article, or claim that may be made by its manufacturer, is not guaranteed or endorsed by the publisher.
